# Bioactivities of Phenolic Compounds from Kiwifruit and Persimmon

**DOI:** 10.3390/molecules26154405

**Published:** 2021-07-21

**Authors:** Young-Mo Kim, Faridah Abas, Yong-Seo Park, Yang-Kyun Park, Kyung-Sik Ham, Seong-Gook Kang, Martyna Lubinska-Szczygeł, Aviva Ezra, Shela Gorinstein

**Affiliations:** 1 Industry Academic Collaboration Foundation, Kwangju Women’s University, Gwangsan-gu, Gwangju 62396, Korea; bliss0816@kwu.ac.kr; 2 Department of Food Science, Faculty of Food Science and Technology, University Putra Malaysia, Serdang 43400, Selangor, Malaysia; faridah_abas@upm.edu.my; 3 Laboratory of Natural Products, Institute of Bioscience, University Putra Malaysia, Serdang 43400, Selangor, Malaysia; 4 Department of Horticultural Science, Mokpo National University, Muan 534-729, Jeonnam, Korea; ypark@mokpo.ac.kr; 5 Department of Food Engineering, Mokpo National University, Muan 534-729, Jeonnam, Korea; ykpark@mokpo.ac.kr (Y.-K.P.); ksham@mokpo.ac.kr (K.-S.H.); sgkang@mokpo.ac.kr (S.-G.K.); 6 Department of Analytical Chemistry, Faculty of Chemistry, Gdansk University of Technology, 80-233 Gdansk, Poland; martyna.lubinska@pg.edu.pl; 7 Faculty of Medicine, Institute for Drug Research, School of Pharmacy, The Hebrew University of Jerusalem, Jerusalem 9112001, Israel; aviva.friedman-ezra@mail.huji.ac.il

**Keywords:** kiwifruit, persimmon, polyphenols, human serum proteins, quenching properties, biomarkers

## Abstract

Fruit used in the common human diet in general, and kiwifruit and persimmon particularly, displays health properties in the prevention of heart disease. This study describes a combination of bioactivity, multivariate data analyses and fluorescence measurements for the differentiating of kiwifruit and persimmon, their quenching and antioxidant properties. The metabolic differences are shown, as well in the results of bioactivities and antioxidant capacities determined by ABTS, FRAP, CUPRAC and DPPH assays. To complement the bioactivity of these fruits, the quenching properties between extracted polyphenols and human serum proteins were determined by 3D-fluorescence spectroscopy studies. These properties of the extracted polyphenols in interaction with the main serum proteins in the human metabolism (human serum albumin (HSA), α-β-globulin (α-β G) and fibrinogen (Fgn)), showed that kiwifruit was more reactive than persimmon. There was a direct correlation between the quenching properties of the polyphenols of the investigated fruits with serum human proteins, their relative quantification and bioactivity. The results of metabolites and fluorescence quenching show that these fruits possess multiple properties that have a great potential to be used in industry with emphasis on the formulation of functional foods and in the pharmaceutical industry. Based on the quenching properties of human serum proteins with polyphenols and recent reports in vivo on human studies, we hypothesize that HSA, α-β G and Fgn will be predictors of coronary artery disease (CAD).

## 1. Introduction

Antioxidant activities of bioactive compounds for human nutrition and health are directly connected with different fruits, such as exotic and traditional, which are commonly used in daily consumption [1,2]. Many tropical fruits are known, but only a small number is widely consumed [3]. Persimmons and kiwifruits are on the list of the most-used tropical fruits, together with banana, mango, and avocado. Consumption of fruit and its biomarkers of intake are the main indices of a healthy diet [4]. Persimmons (*Diospyros kaki*) are recognized as an outstanding source of biologically active components that exhibit many health benefits, such as antioxidant behavior, radical scavenging activity, antihypertensive and antiatherosclerosis activities [5]. The metabolite profiles related to triglyceride/cholesterol metabolism treated with persimmon were studied [6]. The persistent calyx of *Diospyros kaki* is reported to contain phenolic compounds, including condensed tannins together with phenolic components, estimated in 70% acetone extract [7]. Kiwifruits also exhibit antioxidative, antiproliferative, anti-inflammatory, antimicrobial, antihypertensive, antihypercholesterolemic, neuroprotective and antiobese properties and promote gut health [8]. Nuclear magnetic resonance (NMR) has increasingly become an attractive tool in metabolomics analysis and has been combined with multivariate data analysis such as principal component analysis (PCA) and partial least-squares discriminate analysis (PLS-DA) in order to evaluate the properties of the bioactivity of compounds, especially phenolic ones with antioxidant properties. This approach has been used to identify differences among varieties of foods, the quality of cultivar selection, and taste evaluation [9,10,11,12]. The metabolic profiling of three kiwifruit varieties, including the most consumed Hayward cultivar (*Actinidia deliciosa*), the mini kiwi (*Actinidia arguta*), and the less-known Bidan (*Actinidia eriantha*), and their nutritional component analyses in different development stages were investigated in recent studies [13,14]. In spite of the frequent consumption of kiwifruit (*Actinidia*) and persimmon (*Diospyros kaki*), and also of their belonging to the same category as tropical fruits [3] such as mango (*Mangifera indica*), avocado (*Persea Americana*), dragon fruit (*Hylocereus undatus*), and others, information about metabolites in human studies is unavailable [4,15]. The bioactivity of fruit polyphenols in interaction with human serum proteins is an important factor to characterize their health and nutritional properties [16]. Recent reports conducted preclinical and clinical studies on the polyphenol bioavailability and health benefits of aronia berry consumption [17]. While reports on polyphenol bioavailability have increased, there is still limited knowledge about the dynamics of polyphenol metabolism [18,19,20].

Polyphenols and flavonoids are plant metabolites that interfere with different biological processes in the human metabolism. After absorption, in our case through fruits, they bind to human serum albumin (HSA), the most abundant carrier protein in the blood, which also binds various drugs. The binding of flavonoids to HSA may impact their distribution, influencing the active concentration in the blood. Other human proteins in addition to HSA, such as globulins and fibrinogen, also react with polyphenols. Human proteins, including albumin, α-acid glycoprotein, lipoproteins, fibrinogen and α, β, and γ globulins play an important role in the pharmacokinetic and pharmacodynamic properties of food diets. The polyphenols–protein interaction is reversible in that the polyphenols–protein complex can dissociate and release free polyphenols [21,22]. For the first time, our findings indicated that one of the positive benefits of fruit consumption in patients with coronary artery disease (CAD) was diminishing the production of fibrinogen and its stability, which reduces the potential risk exerted by this protein [23]. Serum albumin is a powerful prognostic marker in patients with cardiovascular diseases [24]. The albumin-to-globulin ratio (AGR) is used as a prognostic marker in acute ischemic cardiovascular events. Investigations were directed to determine whether serum AGR, fibrinogen, and the fibrinogen-to-albumin ratio (FAR) are related to the presence and severity of coronary artery disease [25,26].

Using animal models and humans in vitro and in vivo, it was estimated that various fruits, including persimmon and kiwifruit, contained a high number of potential antioxidants and improved the lipid and antioxidant status. Kiwifruit consumption changes plasma lipids, fibrinogen and insulin resistance in the context of a normal diet [27,28]. Pretreatment of platelets with tannic acid led to significant reductions in soluble fibrinogen binding, supporting the inhibition of diverse platelet activation pathways [29]. Some phenolic acid derivatives displayed anticoagulant activities [30].

In spite of wide information on the properties of fruits, the additional information provided in this study on the in vitro interaction with some metabolites is important to understand the health properties of fruit consumption. Based on our recent results and information, in vitro studies will be performed on persimmons and kiwifruits. The contents of the bioactive compounds in these fruits and their influence on the quenching properties of the main proteins in human metabolism such as HSA, globulins and fibrinogen will be studied using advanced analytical methods, including NMR, fluorescence, and different antioxidant assays.

## 2. Results and Discussion

### 2.1. Identification of Bioactive Compounds in Fruit Extracts

**Figure 1:**
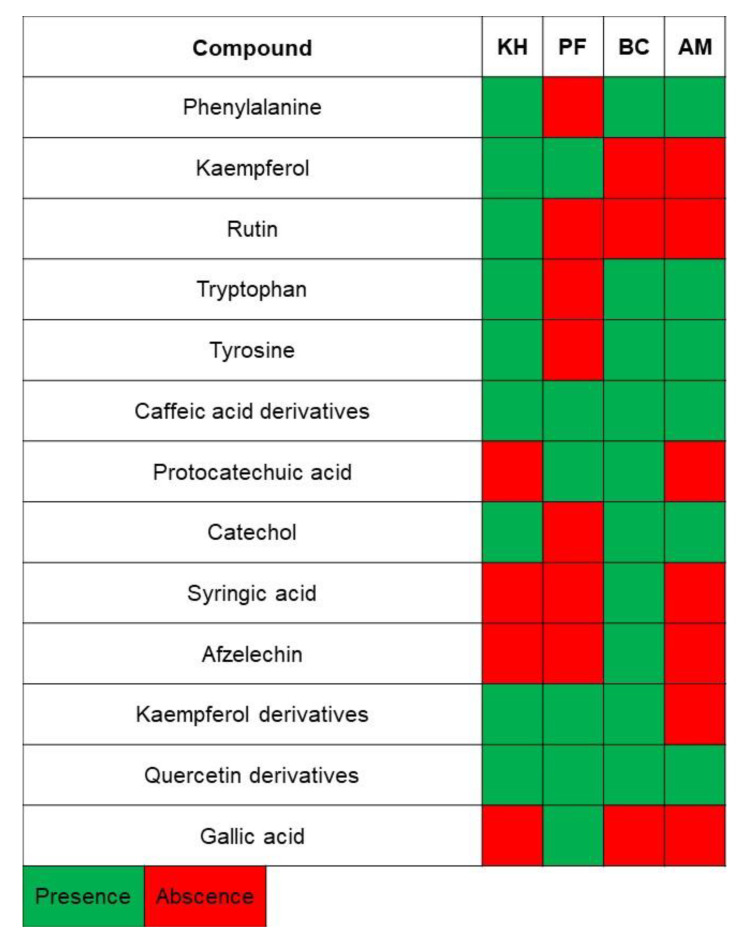
The presence of bioactive compounds in fruit samples. Abbreviations: KH, *Actinidia (A.) deliciosa* cv. Hayward; BC, *A*. *eriantha* cv. Bidan; AM, *A*. *arguta* cv. Cheongsan; PF, *Diospyros kaki* Thunb. cv. Fuyu.

In this study, three different varieties of kiwifruits, such as *Actinidia* (A.) *deliciosa* cv. Hayward (KH), *A. eriantha* cv. Bidan (BC), *A. arguta* Cheongsan (AM) and one cultivar of *Diospyros kaki* Thunb. cv. Fuyu (PF), were subjected to NMR analysis, and the obtained spectra were further evaluated using multivariate data analysis (MVDA). The identified metabolites and their NMR characteristics, as well as their presence in each sample, are listed in Table 1 and Figure 1.

**Figure 2:**
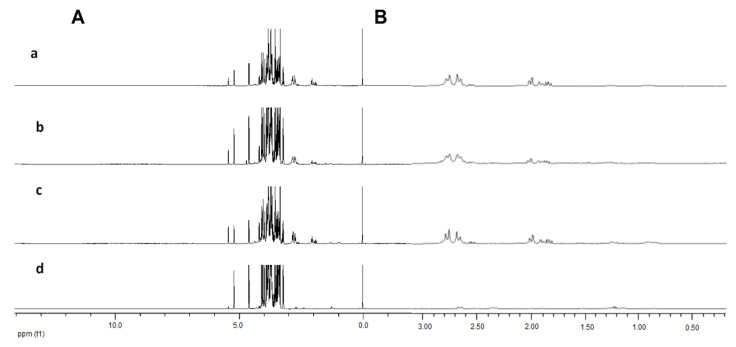
(**A**) The representative ^1^H-NMR spectra in the δ 3.00 to 5.00 range of kiwifruits (**a**) Hayward, (**b**) Bidan, (**c**) *A. arguta* and (**d**) persimmon Fuyu; (**B**) the representative expanded ^1^D-NMR spectra in the δ 0.02 to 3.00 range from top to bottom: Hayward, Bidan, *A. arguta* and persimmon Fuyu.

There are differences in some metabolites of the different fruit samples, which can be detected from the ^1^H-NMR spectra. The principal peak areas of the spectra impart, as an immediate measure of metabolite concentration, permitting whole metabolites to be quantified based on a single internal standard. Different peak intensities can be observed at δ 5.20 (α glucose) and δ 5.40 (sucrose), where persimmon Fuyu showed the lowest concentration of sucrose as compared with the other kiwifruit samples (Figure 2A). It is also worth noting that different levels of peaks can be seen in the aliphatic region (δ 0.50–δ 3.00, Figure 2B). The same trend of intensities was observed in persimmons with the lowest signal compared with the tested kiwifruits. However, visual inspection of ^1^D-NMR does not permit any detection of compounds in the aromatic region (δ 6.00–δ 8.50). The ^1^D-NMR spectra of three different kiwifruits and persimmon, focusing on the region of δ 0.02 to 3.00 and different levels of signal intensities, can be observed in the aliphatic region (Figure 2B). Persimmons demonstrated the lowest intensities compared with kiwifruits.

### 2.2. Principal Component Analysis (PCA) and Multivariate Data Analysis (MVDA)

**Figure 3:**
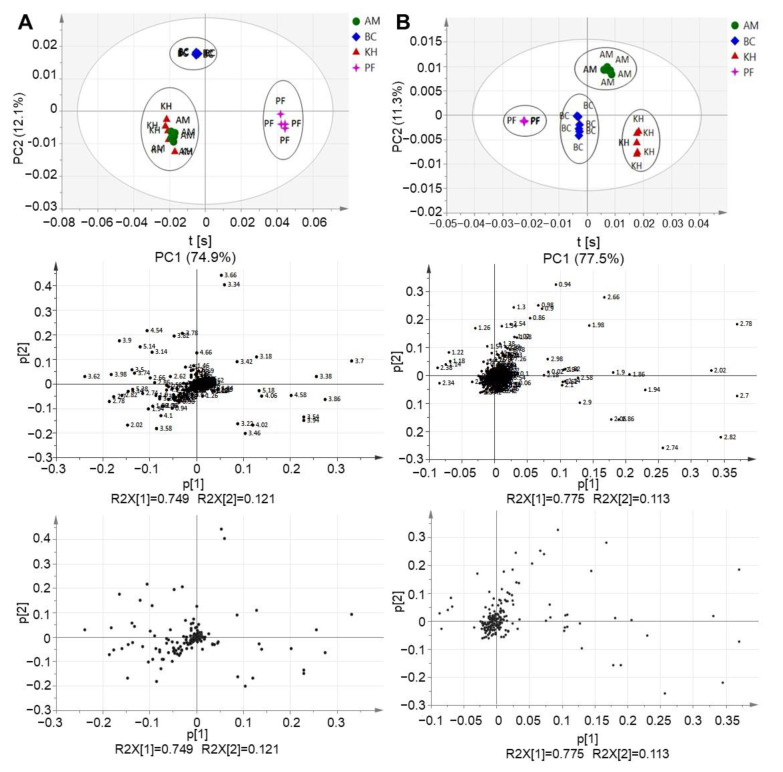
(**A**) Principal Component Analysis (PCA) was performed on the NMR chemical shifts and revealed a significant separation among the samples. PCA score plot of three different kiwifruits: Hayward (KH), Bidan (BC), *A. Arguta* (AM) and persimmon Fuyu (PF). (**B**) PCA score plot after excluding the sugar region of KH, BC, AM and PF.

PCA, as an unsupervised classification method, can be performed without prior knowledge of the data set, simplifying the dimensionality of the numerous variables while virtually sustaining the variances. The outcome of the PCA examination comprised of score plots, which signified the variation of the classes based on the metabolomics similarity, and loading plots, which offers information as to which NMR spectral regions were contributing with respect to the grouping attained in the score plots. Three different clusters were formed between the studied fruit samples (Figure 3A). The persimmon can be discriminated from kiwifruit samples by PC1. Meanwhile, BC can be distinguished from the other two kiwifruit varieties by PC2. However, no separation can be observed between KH and AM. The results (Figure 2A) prompted us to proceed with MVDA by excluding the sugar regions from the ^1^D-NMR spectrum (Figure 3B).

Therefore, as the next step, the sugar region (δ 3.00–δ 5.50) was removed from the binned excel file, and the unsupervised MVDA analysis was then repeated. This was carried out to observe if there was any clustering that could be formed without the interference of sugars in the samples. It is interesting to note that the removal of the sugar region managed to distinguish the KH from AM. Four distinct clusters can be observed where PF and BC are discriminated from AM and KH by PC1. Meanwhile, AM can be separated from the rest of the samples by PC2. Thus, it can be suggested that KH and AM are discriminated from each other without the masking effects of high sugar components (Figure 3B).

**Figure 4:**
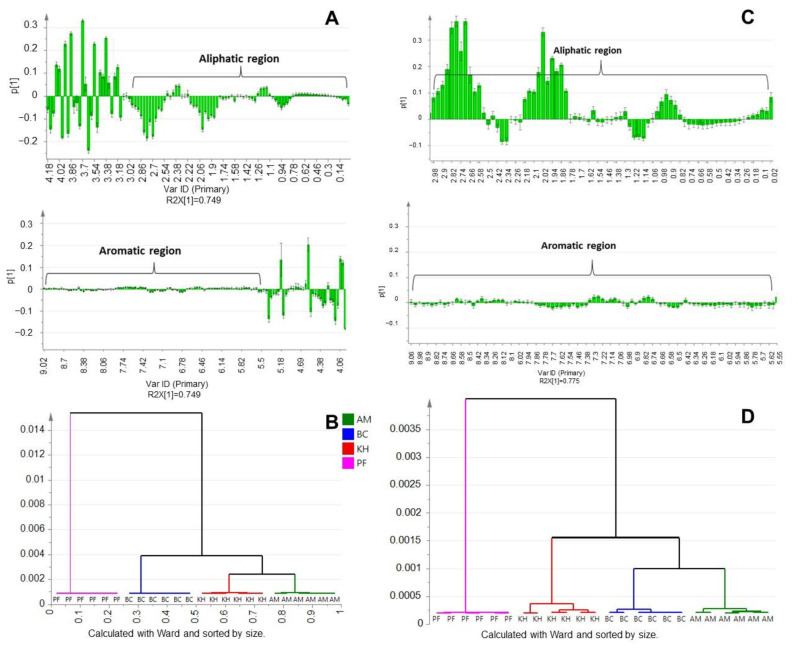
(**A**) PCA loading column plot of three different kiwifruits: Hayward (KH), Bidan (BC), *A. arguta* (AM) and persimmon Fuyu (PF). (**B**) Hierarchical cluster analysis (HCA) for KH, BC, AM and PF, based on group average cluster analysis of the different metabolite components. (**C**) PCA loading column plot for KH, BC, AM and PF after removing sugar regions. (**D**) HCA for KH, BC, AM and PF, based on group average cluster analysis of the different metabolite components after removing sugar regions.

The upper regions of the PCA loading column plot, which corresponded to the persimmon, showed a lower intensity for most of the signals in the aliphatic regions, δ 0.50 to δ 3.00, compared with the lower regions of the model (Figure 4A). This was consistent with the trend demonstrated in the ^1^D-NMR spectra (Figure 2B). The relationship between the three varieties of kiwifruits and the persimmon was further evaluated in the first level of our hierarchical model (Figure 4B). PF was discernible from the other kiwifruit samples, based on different metabolite constitutions. Different results appeared after removing the sugar region (Figure 4C,D).

The obtained results can be compared with our recent data [13], where, in the same three varieties of kiwifruit, phenylalanine, tyrosine, arginine, citric acid, glutamine-hydroxy-L-proline, 4-aminobutyrate (GABA), glutamate, glutamine, quinic acid, actinic acid, shikimate, mannose, syringic acid and afzelechin were detected. The present results on kiwifruit metabolites can be compared with other reports, but there aliphatic part was most prominent. Some investigations were also carried out on parts of fresh kiwifruit or on fruit juices obtained with specific food processing procedures [31]. Similar results for the metabolites in persimmons were obtained in our report [32]. The differences in metabolites among five major Japanese persimmon cultivars were investigated using a nuclear magnetic resonance (NMR)-based metabolomics approach. Among the non-astringent cultivars analyzed, the Matsumotowase-Fuyu cultivar contains ethyl-beta-glycosides, as characteristic components, which may relate to fruit softening. The quantitative metabolomics approach based on broadband WET NMR spectra was mostly discussed in the aliphatic region as well [11,12].

### 2.3. Determination of Bioactive Compounds

**Figure 5:**
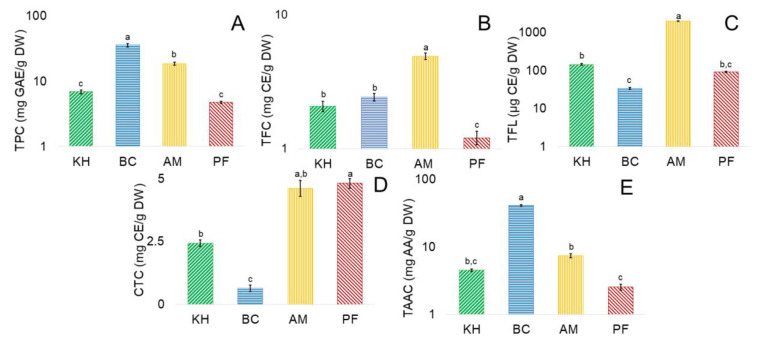
The content of bioactive compounds in investigated fruits. (**A**) Total phenolic content (TPC, mg GAE/g DW); (**B**) total flavonoid content (TFC, mg CE/g DW); (**C**) total flavanols content (TFL, µg CE/g DW); (**D**) condensed tannin content (CTC, mg CE/g DW); (**E**) total ascorbic acid content (TAAC, mg AA/g DW). In figures (**A**–**E**) values are ± SD per gram dry weight (DW); n = 5 samples per cultivar, each subsampled and analyzed five times. Values in bars with different superscript letters are significantly different between the groups of investigated fruit samples in each independent analysis of their quality (*p* < 0.05). Abbreviations: KH, *Actinidia (A.) deliciosa* cv. Hayward; BC, *A. eriantha* cv. Bidan; AM, *A. arguta* cv. Cheongsan; PF, *Diospyros kaki* Thunb. cv. Fuyu; GAE, gallic acid equivalent; CE, catechin equivalent; AA, ascorbic acid; DW, dry weight.

The number of bioactive compounds in kiwifruits and persimmon showed the following ranges (Figure 5).

From the presented results, nearly all bioactive compounds were the highest in BC, following by AM, and similar for KH and PF. Total phenolic content (TPC, mg GAE/g DW) from PF to BC was in the range of 4.74 ± 0.18 to 36.61 ± 2.15, respectively (Figure 5A). Total flavonoid content (TFC, mg CE/g DW) showed the lowest value of 1.21 ± 0.14 for PF and the highest for AM of 4.91 ± 0.28 (Figure 5B). Estimation of total flavanols content (TFL, µg CE/g DW) showed the highest for AM of 1969.81 ± 12.41 and the lowest for BC of 33.63 ± 2.43 (Figure 5C). Condensed tannin content (CTC, mg CE/g DW) changed in the investigated samples from 0.64 ± 0.13 for BC and the highest was for PF of 4.81 ± 0.19 (Figure 5D). Total ascorbic acid content (TAAC, mg AA/g DW) was expressed in PF as the lowest of 2.52 ± 0.27 and with the highest of 40.89 ± 1.18 for BC (Figure 5E). These results are in agreement with some recent reports. *A. eriantha* ‘Bidan’ peeled fruit methanol extracts had a higher TPC of 57.4 in comparison with 12.9 and 6.4 mg GAE/g DW for *A. arguta*, and *A. deliciosa* ‘Hayward’ extracts, respectively [13]. The TPC of six cultivars was in a range of 4.2–14.5 for ethanol extracts and 5.3–16.3 mg GAE/g for water extracts [33]. TPC of three *A. deliciosa* cultivars ranged from 5.3 to 6.6 mg GAE/g DW. *A. arguta*, *A. macrosperma*, and *A. polygama* had TPC of 8.15, 5.57, and 4.71 mg GAE/g DW, respectively [34]. Extracts (70% aqueous acetone) of *A. deliciosa* ‘Hayward’ varied in TPC (479 μg/g DW) [35]. Fresh Korean ‘Hayward’ had a TPC of 8.19 mg GAE/g [36]. TPC and TFC of pulp were 12.21 mg GAE/g DW and 5.92 mg CE/g DW in *Actinidia arguta*. High antioxidant activity was observed (FRAP: 151.41 µmol ferrous sulphate equivalents (FSE)/g DW; DPPH: 12.17 mg TE/g DW). These results emphasize the richness of *A. arguta* fruit pulp to be used in different food products [37]. The TPC of *A. arguta* ‘Chiak’ and ‘Darae No. 2’ ranged from 88 to 113 mg GAE/100 g FW. Hayward cultivar, grown in China, showed 78 mg GAE/100 g FW of TPC; the TFC was evaluated as 10.25 mg CE/100 g FW and DPPH and FRAP were 4.87 and 7.08 µmol TE/g FW [38]. Ethanol and water extracts of the TFC ranged as 1.2–4.3 and 0.6–1.8 mg CE/g DW, respectively [33]. The amount of bioactive compounds varied: total phenolics (mg GAE/g DW) 3.75–8.192–16.52; total flavonoids (mg CE/g DW) 2.11–2.472 and total flavanols (μg CE/g DW) 0.14–0.162 were reported in some recent reports [39,40]. Total flavan-3-ols contents in fourteen kiwifruit cultivars ranged from 96 to 824 μg/g DW [41]. Extraction solvents possibly influenced the solubility of kiwifruit flavonoids [42]. *Actinidia eriantha* [43] is a precious material to study the metabolism and regulation of ascorbic acid (AsA) because of its high content. The other cultivars have relatively high amounts of ascorbic acid, which is shown in Figure 5E and in the published reports [44,45].

### 2.4. Antioxidant Capacities of Investigated Samples

**Figure 6:**
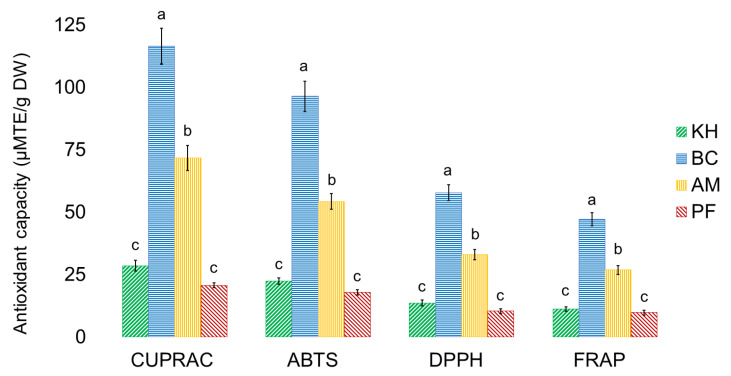
Antioxidant capacities in kiwifruits and persimmons. Values are ± SD per gram of dry weight (DW); *n* = 5 samples per cultivar, each subsampled and analyzed 5 times. Values in bars with different superscript letters are different between the groups of investigated fruit samples in each independent analysis of their quality (*p* < 0.05). Abbreviations: KH, *Actinidia (A.) deliciosa* cv. Hayward; BC, *A. eriantha* cv. Bidan; AM, *A. arguta* cv. Cheongsan; PF, *Diospyros kaki* Thunb. cv. Fuyu; CUPRAC, Cupric reducing antioxidant capacity; ABTS, 2, 2-Azino-bis (3-ethyl-benzothiazoline-6-sulfonic acid) diammonium salt; DPPH, 1, 1-Diphenyl-2-picrylhydrazyl method; FRAP, Ferric-reducing/antioxidant power; TE, Trolox equivalent.

The antioxidant capacities of investigated samples are shown in Figure 6.

The lowest values of CUPRAC, ABTS, DPPH, FRAP (µM TE/g DW) were 20.72 ± 1.23, 17.96 ± 1.02, 10.45 ± 0.35, 9.86 ± 0.61 for PF, and the highest values were 116.63 ± 7.21, 96.48 ± 6.14, 57.87 ± 3.12, 47.37 ± 2.05 (Figure 6). These results are similar to some reports. ‘Hayward’ water extracts had higher FRAP, ABTS, CUPRAC and DPPH values than the investigated methanol extracts [33]. *A. eriantha* ‘Bidan’ extracts had higher DPPH, ABTS, FRAP and CUPRAC values than *A. arguta* and *A. deliciosa* ‘Hayward’ [13,46]. TPC was the greatest antioxidant contributor in the DPPH and FRAP assays, which is in line with other findings [38], as well as with ABTS and CUPRAC (Figure 6). Diversity in the results of bioactive metabolites depends on the varieties and plant parts, extraction, analytical and processing methods, and this affects the physicochemical and biological properties of kiwifruit-derived ingredients [47,48]. The results obtained in this research can be compared with recent reports. Apart from the treatment used, changes in the content of metabolites are also affected markedly by the persimmon variety. Soluble tannins were 23.8 ± 4.3 and 14.3 ± 1.6; soluble non-tannins were 17.4 ± 2.4 and 15.9 ± 0.5; and total phenolic compounds were estimated as 89.1 ± 5.8 and 78.6 ± 4.5, expressed as g of epicatechin equivalents (EE)/kg DW for Rojo Brillante and Kaki Tipo, respectively. Gallic acid was the predominant phenolic compound found in the Rojo Brillante variety (0.953 mg/100 g), whereas the concentration of *p*-hydroxybenzoic acid was higher in the Triumph variety (0.119 mg/100 g). The antioxidant activity values ranged from 1.280 ± 0.069 to 8.865 ± 0.056 µmol TE/g when measured by ABTS, 0.458 ± 0.05 to 2.633 ± 0.03 µmol TE/g when measured by DPPH, and 0.206 ± 0.01 to 0.965 ± 0.005 µmol TE/g when the FRAP method was used. The greatest ABTS scavenging capacity was detected in Rojo Brillante (6.572 µmol TE/g), while the lowest was found in the non-astringent variety Triumph (41.484 µmol TE/g). The antioxidant activities of all extracts determined as DPPH radical scavenging ability ranged from 2.633 to 0.458 µmol TE/g. The ferric-reducing antioxidant power of Rojo Brillante and Triumph extracts was similar to the DPPH scavenging activity and ABTS scavenging capacity, with higher values for the astringent variety (0.965 ± 0.005 µmol TE/g). In general, the astringent variety (Rojo Brillante) showed much higher antioxidant activity than the non-astringent variety (Triumph) for both ABTS (6.572 µmol TE/g and 1.484 µmol TE/g, respectively), DPPH (2.417 µmol TE/g and 0.492 µmol TE/G, respectively), and FRAP assay (0.731 µmol TE/g and 0.242 µmol TE/g, respectively). The Rojo Brillante variety also had the highest values of total phenol content, as measured by the Folin method (380.786 µg GAE/g and 81.568 µg GAE/g, respectively). For persimmon samples, the results were similar to those of other authors, which ranged from 1027.03 to 1667.65 µmol/kg, and the concentrations very close to those reported in our study, although very few papers discussed antioxidants in the fruits considered in this study. Our results are similar and consistent with the data from other research reports, which investigated diverse persimmon genotypes [48,49,50,51].

### 2.5. Quenching Properties of Phenolic Compounds of Investigated Fruits with Human Serum Proteins

**Figure 7:**
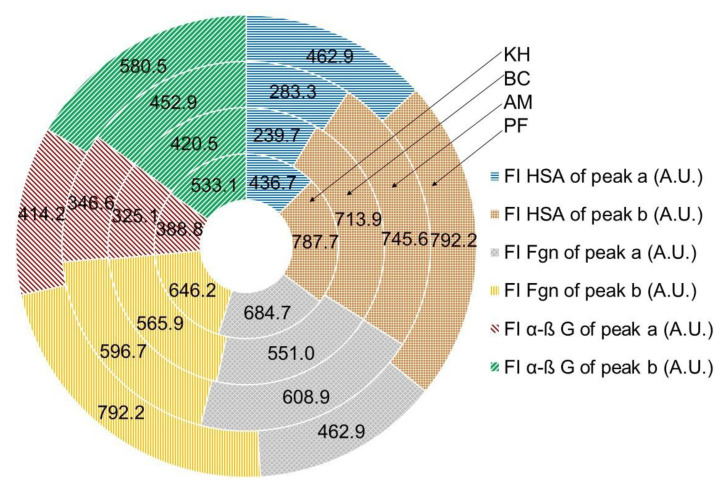
Fluorometric measurements in three-dimensional fluorescence analysis (3D-FL) of kiwifruit and persimmon extracts after interaction with HSA, Fng and α-β-G. Abbreviations: KH, *Actinidia (A.) deliciosa* cv. Hayward; BC, *A. eriantha* cv. Bidan; AM, *A. arguta* cv. Cheongsan; PF, *Diospyros kaki* Thunb. cv. Fuyu; HSA, human serum albumin, α-β G, α-β-globulin; Fgn, fibrinogen, FI, fluorescence intensity, A.U, arbitral units. The values of fluorescence intensity for used human serum proteins before interaction with extracted proteins were the following: FI HSA of peak **a** (A.U.) = 545.9; FI HSA of peak **b** (A.U.) = 814.9; FI Fgn of peak **a** (A.U.) = 877.4; FI Fgn of peak **b** (A.U.) = 809.6; FI α-β G of peak **a** (A.U.) = 457.3; FI α-β G of peak **b** (A.U.) = 661.1. The locations of peaks **a** and **b** are shown in Figure 8 and Figure 9 (for interpretation of the references to color in this figure legend, the reader is referred to the web version of this article).

**Figure 8:**
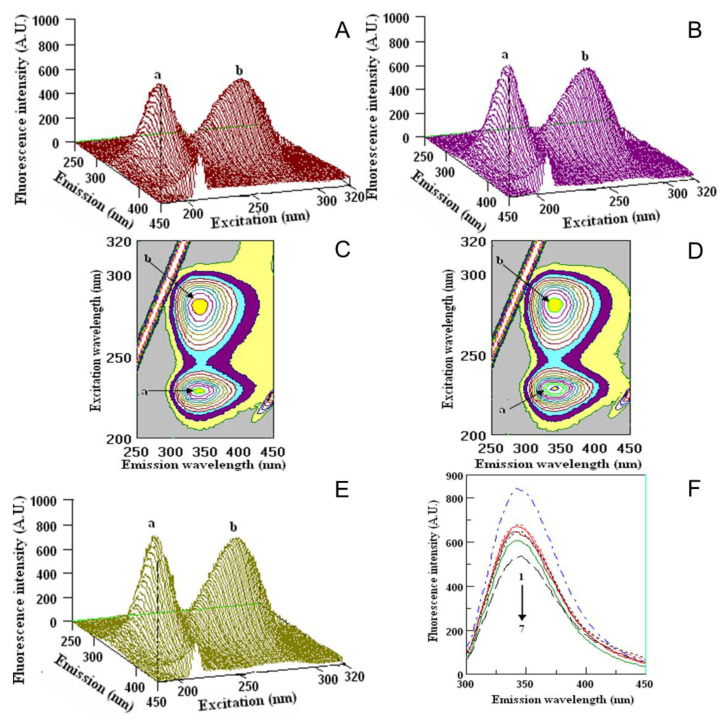
Fluorometric measurements in three-dimensional fluorescence analysis (3D-FL) of kiwifruit and persimmon extracts after interaction with fibrinogen. (**A**,**B**) 3D-FL of KH and PF after interaction with Fng; and (**C**,**D**) their cross images. (**E**) Fibrinogen. (**F**) Spectral data of two-dimensional fluorescence measurements (2D-FL) of fruit extracts and Fng from the top: 1, 2, 3, 4, 5, 6, 7, Fgn (FI = 834.71 A.U.), Fgn + quercetin (FI = 680.42 A.U.), Fgn + caffeic acid (FI = 671.79 A.U.), Fgn + KH (FI = 649.13 A.U.), Fgn + PF (FI = 648.29 A.U.), Fgn + catechin (FI = 606.17 A.U.), Fgn + tannic acid (FI = 538.23 A.U.). Abbreviations: KH, *Actinidia (A.) deliciosa* cv. Hayward; PF, *Diospyros kaki* Thunb. cv. Fuyu; Fgn, fibrinogen, FI, fluorescence intensity, A.U, arbitral units. The locations of peaks **a** and **b** are shown in Figure 7 and Figure 9 (for interpretation of the references to color in this figure legend, the reader is referred to the web version of this article).

**Figure 9:**
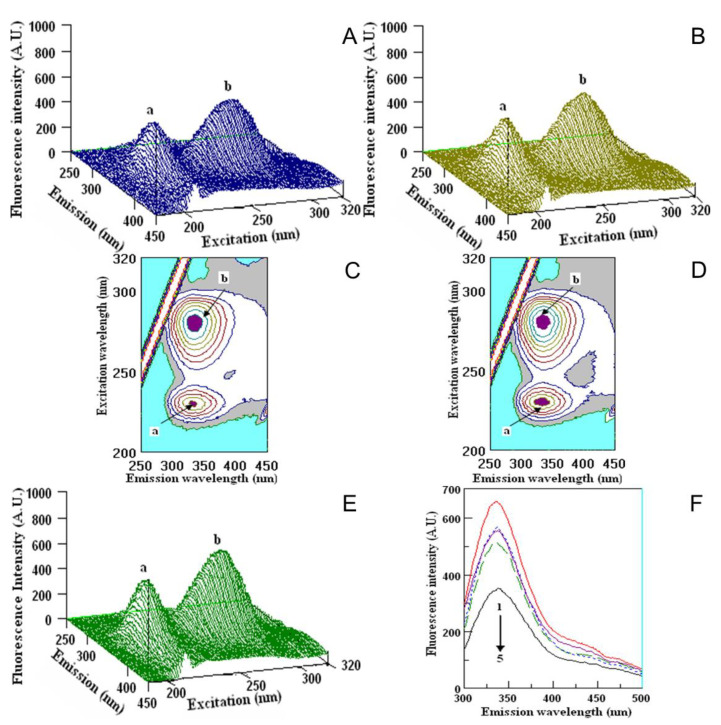
Fluorometric measurements in three-dimensional fluorescence analysis (3D-FL) of kiwifruit and persimmon extracts after interaction with globulin. (**A**,**B**) 3D-FL of KH and PF after interaction with α-β G, and (**C**,**D**) their cross images. (**E**) α-β G; (**F**) spectral data of two-dimensional fluorescence measurements (2D-FL) of fruit extracts and α-β G from the top: 1, 2, 3, 4, 5, α-β G (FI = 658.57.71 A.U.), α-β G + PF (FI = 567.49 A.U.); α-β G + quercetin (FI = 554.04 A.U.); α-β G + KH (FI = 515.68 A.U.), α-β G + tannic acid (FI = 353.19 A.U.). Abbreviations: KH, *Actinidia (A.) deliciosa* cv. Hayward; PF, *Diospyros kaki* Thunb. cv. Fuyu; α-β G, α-β-globulin; FI, fluorescence intensity; A.U, arbitral units. The locations of peaks **a** and **b** are shown in Figure 7 and Figure 9 (for interpretation of the references to color in this figure legend, the reader is referred to the web version of this article).

The interactions of fruit extracts with human serum albumin (HSA), fibrinogen (Fgn) and α, β-globulin (α, β-G) are shown in Figure 7, Figure 8 and Figure 9.

The interaction with the above serum proteins and extracted fruit polyphenols is evaluated by the changes in the fluorescence intensity of the proteins. The changes appeared mostly in the position and value of peak **a**. The values in the fluorescence intensity (F.I.) of peak **a** in HSA with fruit extracts were the highest for PF (462.9 ± 6.9 A.U.) and the lowest for BC (239.7 ± 5.1). Small changes appeared in the position and value of peak **b**. The fluorescence intensity of HSA with fruit extracts was the highest for PF (792.2 ± 9.3 A.U.) and the lowest for BC (713.9 ± 9.9 A.U.), in comparison with the initial ones (Figure 7). The FI of peak **a** of Fgn after interaction with fruit extracts was the highest for PF (775.2 ± 8.2 A.U.) and the lowest for BC (551.0 ± 6.9). Lower changes in comparison with peak **a** appeared in the position of peak **b**: the highest peak was measured for PF (700.2 ± 7.5 A.U.) and the lowest one was measured for BC (565.9 ± 5.9 A.U.). The exact locations of peaks **a** and **b** during the interaction of KH and PF are presented in Figure 7 and Figure 8.

The images of the interaction of Fgn with KH and PF (Figure 8A–D) and the Fgn (Figure 8F) showed the maximum peaks **a** and **b** and their locations. The comparison of the values of fluorescence intensity of the native Fgn (Figure 8F, line 1 from the top) showed that the lowest value was obtained by its interaction with tannic acid (Figure 8F, line 7). In 2D-FL, the values of fluorescence intensities with extracts of KH and PF were nearly similar (Figure 8F, lines 4 and 5).

The fluorescence measurements with serum globulin and fruit extracts are presented in Figure 7 and Figure 9. Peak **a** of α-β-globulin (α-β G) after interaction with fruit extracts was the highest for PF (414.2 ± 7.4 A.U.) and the lowest for BC (325.1 ± 3.9). Lower changes appeared in the position of peak **b**: the highest peak was measured for PF (580.5 ± 7.5 A.U.) and the lowest was measured for BC (420.5 ± 3.3 A.U.). The exact locations of peaks **a** and **b** during the interaction with KH and PF are presented in Figure 7 and Figure 9.

The images of the interaction of α-β G with KH and PF (Figure 9A–D) and the α-β G (Figure 9E) showed the maximum of peaks **a** and **b** and their locations. The comparison of the values of fluorescence intensity of the native α-β G (Figure 9F, line 1 from the top) showed that the lowest value was obtained by its interaction with tannic acid (Figure 9F, line 5). In 2D-FL, the changes in fluorescence intensities with extracts of KH and PF were almost similar to Fgn, but showed slightly different values in reaction with globulin, and KH was more reactive than PF (Figure 9F, lines 4 and 2, respectively).

Albumin, fibrinogen, lipoproteins and α, β, and γ globulins play an important role in the pharmacokinetic properties of food nutriments. Globulins make up 35% of plasma proteins and are used in the transport of ions, drugs and lipids [21,22]. It was important to compare the standard metabolites with the polyphenol extracts. The polyphenols-protein interaction is reversible in that the polyphenols–protein complex can dissociate and release free polyphenols. Polyphenols and their metabolites rapidly exchange between free and bound forms within the circulation. Reversible binding to plasma proteins may have consequences for the delivery of the polyphenols and their metabolites to cells and tissues [21,22].

**Figure 10:**
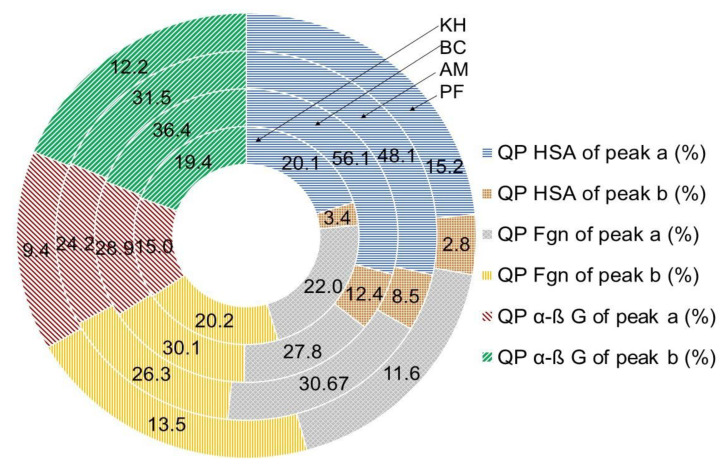
The quenching properties (QP, %) of fruit-extracted polyphenols based on interaction with human serum proteins and fluorometric measurements. Abbreviations: KH, *Actinidia (A.) deliciosa* cv. Hayward; BC, *A. eriantha* cv. Bidan; AM, *A. arguta* cv. Cheongsan; PF, *Diospyros kaki* Thunb. cv. Fuyu; HSA, human serum albumin; α-β G, α-β-globulin; Fgn, fibrinogen. The locations and values of peaks **a** and **b** are shown in Figure 7, Figure 8 and Figure 9 (for interpretation of the references to color in this figure legend, the reader is referred to the web version of this article).

The quenching properties (%) between HSA and polyphenols for PF and BC were in the range of 15.2 ± 0.9 to 56.15.8, calculated by peak **a** and 2.8 ± 0.1 to 12.4 ± 1.1, according to peak **b**, respectively. Different values of quenching properties (%) were calculated with fibrinogen interaction: PF and BC were in the range of 11.6 ± 1.1 to 37.8 ± 2.4, calculated by peak **a**, and 13.5 ± 1.1 to 28.9 ± 1.2, according to peak **b**, respectively, and globulin showed 9.4 ± 0.9 and 37.8 ± 2.4, calculated by peak **a**, and 12.2 ± 1.1 and 36.4 ± 2.4, according to peak **b**, respectively (Figure 10).

The presented data varied between the varieties and the used serum proteins, but showed the same correlation between samples, where BC was the strongest and KH and PF were in the same range of their bioactivities (Figure 7, Figure 8, Figure 9 and Figure 10).

In the present report, we have used a simplified measure to show only the decrease in fluorescence emission after the addition of a single concentration of ligands. This can be regarded as a relative measure of quenching, providing that the inner filter is similarly negligible within the series of ligands. Thus, the % decrease of fluorescence represents the fraction of the binding sites of the protein occupied by the ligand, rather than the fraction of the total ligand bound to the protein [52].

The obtained metabolite results of persimmon samples showed relatively high amounts of tannins in comparison with the investigated three samples of kiwifruits and are in line with several reports [5,6,11,12]. The estimation of quenching of serum proteins such as fibrinogen (Figure 8F, line 8) and globulin with tannic acid (Figure 9, line 5) are in full agreement with the amount of tannic acid in persimmon samples. Based on these results, PF showed nearly the same quenching properties as KH. The presently determined high quenching properties of persimmon in vitro with relatively new metabolite indices such as fibrinogen and globulin, showing protective action and preventing CAD, are in line with some reported in vitro and in vivo studies. Evaluation of the prognostic significance of changes in serum albumin levels among patients that underwent percutaneous coronary intervention (PCI) showed that a decrease in albumin levels following PCI is an independent prognostic marker of worse long-term outcomes [24]. It was found that, similar to traditional risk factors, plasma fibrinogen and albumin levels showed a close relation with the presence and severity of CAD [25]. The fibrinogen-to-albumin ratio index is a valuable biomarker associated with ST- elevation myocardial infarction and may be useful in the prediction of the long-term prognosis of patients with such diseases [26]. So, following the recent reports on humans discussed above and the results of the supplementation of fruits, it was shown that the triglyceride (TG)/cholesterol profile depended on the treatment of persimmon water extracts, and tannin-enriched persimmon concentrate stimulated hypocholesterolemic actions [6,53,54]. Similar action was obtained by a combination of *Diospyros kaki* fruit and *Citrus unshiu* peel mixture as a potential therapeutic agent for treating nonalcoholic fatty liver disease with the remarkable growth of obesity [55]. Variation in tannin amount depends on the cultivars of persimmon, even in co-products from cvs. Rojo Brillante’ and Triumph’ persimmon juice extraction processed to obtain flours rich in the main metabolites, such as sugars, organic acids, tannins, and bioactive compounds, suggesting their use as a functional ingredient with antioxidant properties in different food products [56]. Similar results for the quenching properties of kiwifruit polyphenols were obtained in the present study. As such, the health properties of kiwifruit polyphenols results shown in Figure 7, Figure 8, Figure 9 and Figure 10 are in compliance with previous studies, where the intervention of green kiwifruit effectively lowered the total cholesterol (TC) and increased the high-density lipoprotein cholesterol (HDL) concentration in hypercholesterolaemic and healthy individuals. It was proven that the bioavailability of polyphenols depends on physicochemical stability, complex formation, food interaction, gastrointestinal absorption, and hepatic and gut metabolism [19]. Consumption of fruits influences the total cholesterol, LDL, HDL, proteins, lipid peroxidation and oxidative stress biomarkers [17,18,20]. Consumption of at least one kiwi/week is associated with lower plasma concentrations of fibrinogen and improved plasma lipid profile in the context of a normal diet and regular exercise [28,57,58]. Similar results were reported on antihypercholesterolemia Male Wistar rats when 1% cholesterol-enriched diet induced-hypercholesterolemia improved liver somatic index and lipid profiles after supplementing with 5% lyophilized Polish grown kiwifruit. *A. arguta* ‘Geneva’, ‘Anna’, and ‘Weiki’ showed the most significant results [46,59]. Cellular antioxidant activity (CAA) assays, combined with clinical trials, will more effectively identify antioxidant phytochemicals in fruits that can be used as dietary additives or drugs for human health. In spite of advanced methods in the determination of antioxidant activities, in vitro studies of the interaction of polyphenols with human serum proteins, in vivo experiments, or clinical trials are still required to verify the efficacious activity when fruit polyphenols are used as dietary supplements or drugs to combat oxidative stress [60].

## 3. Materials and Methods

### 3.1. Chemicals and Materials

The chemicals 2,4,6,-tripyridyl-s-triazine (TPTZ), 6-Hydroxy-2,5,7,8-tetra-methylchroman-2-carboxylic acid (Trolox), 1,1-diphenyl-2-picrylhydrazyl (DPPH), lanthanum(III) chloride heptahydrate, CuCl_2_·2H_2_O, 2,9-dimethyl-1,10-phenanthroline (neocuproine), 2,2-azino-bis(3-ethylbenzothiazloine-6-sulphonic acid) (ABTS) radical cation, ferric chloride, caffeic acid, quercetin, tannic acid, catechin, human serum albumin (HSA), fibrinogen, globulin, phosphate buffer and Folin-Ciocalteu reagent (FCR) were purchased from Sigma (St. Louis, MO, USA) and Fluka Chemie Gm bH, Buchs, Switzerland. All NMR chemicals, including 3-trimethylsilylpropanoic acid (TSP), potassium phosphate monobasic (KH_2_PO_4_), methanol-d4 (CD_3_OD, 99.8%), sodium deuterium oxide (NaOD), and deuterium oxide (D_2_O, 99.9%), were purchased from Merck (Darmstadt, Germany).

### 3.2. Sampling and NMR Metabolomics

Three batches of organic kiwifruits, including *Actinidia (A.) deliciosa* cv. Hayward (KH), *A. eriantha* cv. Bidan (BC), *A. arguta* Cheongsan (AM) and one batch of *Diospyros kaki* Thunb. cv. Fuyu (PF), were collected in different commercial orchards from Boseong and Muan counties, Jeonnam and Wonju-si, Gangwon-do provinces, South Korea [61]. Each batch was composed of 25 fruits, about two kg in weight. The cultivars reached the commercial maturity stage. The samples were washed with tap water and dried. The fruits were fractionated into an edible fraction (pulps), peels and seeds. Only for PF, 5–8 seeds were separated from pulps. Their edible parts were prepared manually without using steel knives. The peeled fruits (pulps) were weighed, chopped and homogenized in liquid nitrogen in a high-speed blender (Silex professional model, Hamilton Beach, Virginia, USA). A weighed portion (50–100 g) was then lyophilized for 48 h (Virtis model 10–324, Midland, ON, Canada), and the dry weight was determined. The samples were ground to pass through a 60-mesh sieve and stored at −20 °C until the bioactive substances were analyzed.

The proton (^1^H) and two-dimensional (2D) J-resolved NMR procedure was carried out according to the previously reported protocols with small modifications. The extraction of samples was carried out by transferring 100 mg of each sample into a 2 mL Eppendorf tube, followed by the addition of 375 µL of both CD_3_OD solvent and KH_2_PO_4_ buffer in D_2_O (pH 6.0) containing 0.1% TSP. The solution was then vortexed for 1 min before being subjected to sonication for 15 min at a controlled temperature. To get a clear supernatant, the mixture was afterward centrifuged at rpm for 10 min, and 600 µL of it was pipetted to a NMR tube prior to analysis. The ^1^H-NMR analysis was performed at 25 °C on an INOVA 500 MHz spectrometer (Varian Inc., Palo Alto, CA, USA). For each sample, the required time was 3.53 min, recording 64 scans with an acquisition time, a pulse width, and a relaxation delay of 220 s, 3.75 ms, and 1.0 s, respectively. These settings were for presaturation prior to ^1^H-NMR, which is required to suppress the water signal using low power selective irradiation. In addition, the spectral width of the recorded spectra was 20 ppm. The processing for all spectra, including phasing and baseline corrections, was performed manually with Chenomx software (Version 6.2, Edmonton, AB, Canada). Moreover, the 2D J-resolved was conducted to endorse metabolite identification [13,32,62].

### 3.3. Determination of Bioactive Compounds

The detailed procedures of the extraction, determination of bioactive compounds and their antioxidant capacities were described in our very recent reports [52,63,64]. For polyphenol extraction, the freeze-dried powders of investigated samples were immersed in absolute methanol (1/10 *w*/*v*). The filtrate was collected three times with constant stirring of the mixture at every 24 h interval of a 72 h total collection period at room temperature. The extract was then concentrated under reduced pressure at 45 °C using a vacuum rotary evaporator.

A Folin–Ciocalteu assay was used for the determination of total polyphenol content (TPC) in methanol fruit extracts of 0.25 mL with 1 mL of Folin–Ciocalteu reagent (Sigma, St. Louis, MO, USA). Then, 0.75 mL of 1% sodium carbonate was added. Absorbance of the mixture was measured on a Hewlett-Packard model 8452A spectrophotometer (Hewlett-Packard, Rockville, MD, USA) at 750 nm. The results were calculated in mg gallic acid equivalents (GAE) per g DW [65]. Total flavonoid content (TFC, mg catechin equivalents (CE) per g DW) was measured at 510 nm after extraction with 5% NaNO_2_, 10% AlCl_3_xH_2_O and 1 M of NaOH [66]. The absorbance of total flavanols (TFL, µg CE per g DW) was measured at 640 nm following the *p*-dimethylaminocinnamaldehyde (DMACA) method: 1 mL of DMACA solution was added to 0.2 mL of fruit extracts [67]. Condensed tannin content (CTC, mg CE per g DW) was estimated by spectrophotometric measurements in the mixture of methanol fruit extracts and the addition of 4% methanol vanillin solution. Absorbance was measured at 500 nm after the end of the reaction [68]. Total ascorbic acid content (TAAC, mg ascorbic acid (AA) per g DW) was evaluated in water fruit extracts, where 100 mg of the freeze-dried sample was extracted with 5 mL water. Then, CUPRAC method was conducted, and formed bis (Nc)-copper (I) chelate was determined spectrophotometrically at 450 nm [69].

### 3.4. Determination of Antioxidant Capacities

Total antioxidant capacity was determined by the following assays, which are also described in our recent reports [52,63,64].

Cupric reducing antioxidant (CUPRAC) assay is based on utilizing the copper (II)−neocuproine reagent as the chromogenic oxidizing agent. Absorbance at 450 nm was measured in a mixture of [Cu (II)-Nc] and NH_4_Ac buffer solution and fruit methanol extracts [70].

2,2′-azino-bis(3-ethyl-benzothiazoline-6-sulfonic acid) diammonium salt (ABTS•+) was generated by the interaction of ABTS (7 mM) and K_2_S_2_O_8_ (2.45 mM). This solution was diluted with methanol and measured at 734 nm [71]. Scavenging free radical potentials were tested in a methanolic solution (3.9 mL) of 1, 1-diphenyl-2-picrylhydrazyl (DPPH) with the sample extracts in methanol (0.1 mL) [72]. Ferric-reducing/antioxidant power (FRAP) assay measures the ability of the antioxidants in the investigated samples to reduce ferric-tripiridyltriazine (Fe^3+^-TPTZ) to a ferrous form (Fe^2+^) [73]. All values of antioxidant capacities were expressed in µM trolox equivalent (TE)/g DW.

### 3.5. Fluorometric Studies

The profiles and properties of polyphenols in methanol extracts were determined by two (2D-FL) and three-dimensional (3D-FL) fluorescence (model FP-6500, Jasco spectrofluorometer, serialN261332, Tokyo, Japan). The 2D-FL measurements were taken at emission wavelengths from 310 to 500 nm and at excitation of 295 nm. The 3D-FL was measured at emission wavelengths between 200 and 795 nm and the initial excitation wavelength at 200 nm. For comparison of the obtained results, caffeic acid, quercetin, tannic acid and catechin were used [36]. The quenching properties of phenolic compounds in kiwifruit and persimmon extracts to human serum albumin (HSA), fibrinogen and globulin were evaluated by 2D and 3D-FL. For the fluorescence measurements, 3.0 mL of 1.0 × 10^−5^ mol/L HSA were prepared in 0.05 mol/L Tris–HCl buffer (pH 7.4), containing 0.1 mol/L NaCl. Fibrinogen and globulin stock solutions were made by dissolving in phosphate buffer (10 mM, pH 7.4) to obtain a concentration of 20 µM. Standards phenolic solutions, such as tannic acid, quercetin, catechin, and caffeic acid stock solution, were prepared daily by dissolving at a concentration of 10 mM in methanol and then diluting with 10 mM phosphate buffer at pH 7.4. Samples were prepared by mixing fibrinogen, fruit extracts and standards of phenolic compound solutions in varying proportions. The highest resulting methanol concentration was about 1%, which had no appreciable effect on protein structure. All samples were kept at 4 °C prior to the analysis. The initial fluorescence intensities of HSA, globulin and Fgn were measured before interaction with the investigated samples and pure substances and after interaction with the samples (quenching of the fluorescence emission of proteins, in our case of HSA, globulin and fibrinogen and polyphenols of fruits). As mentioned above, changes in the fluorescence intensities were used in the estimation of quenching activities. [52,63,64].

### 3.6. Data Analysis

NMR data analysis followed the reported procedure [74]. The conversion of ^1^H-NMR spectra to an ASCII file using Chenomx software was carried out prior to multivariate data analysis (MVDA) and performed using SIMCA-P+ version 13.0 (Umetrics AB, Umeå, Sweden). This analysis consists of the exclusion of the residual water (4.70–4.90 ppm) and methanol (3.23–3.34 ppm) signals range. Next, all spectra were scaled to TSP and bucketed to bins with a width of 0.04 ppm, forming a spectral region of 0.52–9.99 ppm. The binned integral of ^1^H-NMR data were then subjected to principal component analysis (PCA), which was applied to clearly differentiate the ^1^H-NMR spectra of the kiwifruit and persimmon samples. The Pareto method was also used for scaling purposes to ensure the same importance was given to all x variables in the analyses. All obtained data were calculated on the basis of statistical analysis of Duncan’s multiple range test. Values are means ± SD per gram of dry weight (DW) of 25 measurements, representing the commercial maturity status of fruits and their replicates. Five biological replications of five extracts from each cultivar were performed. To determine the statistical significance as a 95% interval of reliability, one-way analysis of variance (ANOVA), was used.

## 4. Conclusions

We obtained relatively high amounts of antioxidants in the raw pulp of investigated fruits and high quenching properties of fruit extracts in comparison with pure metabolites. The addition of such fruits to generally accepted diets could be beneficial for hyperlipidemic, especially hypertriglyceridemic, patients suffering from coronary atherosclerosis. We expect that HSA, Fgn and α-β G will serve as predictors of cardiovascular events.

## Figures and Tables

**Table 1:** The identified bioactive compounds and their characteristics.

No.	Compound	CAS	Structure	δH(ppm), Multiplicity, J Value (Hz)
**1**	Phenylalanine	63-91-2	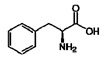	7.40, m (2H) 7.35, m 7.30, d, 7.4 (2H)
**2**	Kaempferol	520-18-3	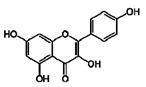	8.01, d, 8.0 6.95, d, 8.0 6.32, br d (small d) 6.10, br d (small d)
**3**	Rutin	153-18-4	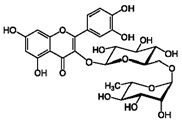	7.65, d, 2.0 7.60, dd, 6.82, d, 8.5 6.38, d, 6.19, d, 1.05, d, 7.0 4.51, br s (small d) 5.05, d, 8.0
**4**	Tryptophan	73-22-3	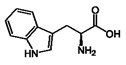	7.70, d, 8.0 7.54, d, 8.0 7.20, t, 7.0
**5**	Tyrosine	60-18-4	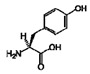	3.94, m 7.15, d, 8.0 6.82, d, 8.0
**6**	Caffeic acid derivatives		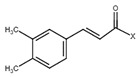	7.57, d, 13.0 7.28, br s (small d) 7.22, d, 8.0 6.95, d, 8.0 6.55, d, 13.0
**7**	Protocatechuic acid	99-50-3	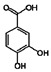	7.39, br s (small d) 7.35, br d (dd), 8.0 6.92, d, 8.0
**8**	Catechol	120-80-9	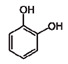	6.77–6.84, m 4.52, d, 7.20 2.94, dd, 15.7, 6.2 2.47, dd, 15.0, 8.0
**9**	Syringic acid	530-57-4	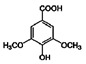	7.26, s, 2H 3.89, s
**10**	Afzelechin	2545-00-8	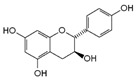	2.83; 2.80; 2.79, dd,15.6, 4.8 2.68, dd 6.85, d, 8.0 (2H) 7.17, d, 8.0 (2H)
**11**	Kaempferol derivatives		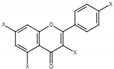	6.97, d, 2.7 6.46, d, 2.7
**12**	Quercetin derivatives		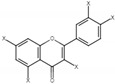	7.52, d, 3.5 6.66, d, 3.5
**13**	Gallic acid	149-91-7	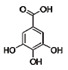	7.01 (s)

## Data Availability

The data presented in this study are available on request from the Corresponding author. The data are not publicly available due to privacy reasons.

## Sample Availability

Samples of the compounds are not available from the authors.
